# Smart biomaterials for enhancing cancer therapy by overcoming tumor hypoxia: a review

**DOI:** 10.1039/d2ra06036a

**Published:** 2022-11-25

**Authors:** Samar A. Salim, Taher A. Salaheldin, Mohamed M. Elmazar, A. F. Abdel-Aziz, Elbadawy A. Kamoun

**Affiliations:** Nanotechnology Research Center (NTRC), The British University in Egypt (BUE) El-Sherouk City Cairo 11837 Egypt e-b.kamoun@tu-bs.de badawykamoun@yahoo.com +20-1283320302; Biochemistry Group, Dep. of Chemistry, Faculty of Science, Mansoura University Egypt; Department of Medicine, Case Western Reserve University School of Medicine Cleveland OH 44106 USA; Faculty of Pharmacy, The British University in Egypt (BUE) El-Sherouk City Cairo 11837 Egypt; Polymeric Materials Research Dep., Advanced Technology and New Materials Research Institute (ATNMRI), The City of Scientific Research and Technological Applications (SRTA-City) New Borg Al-Arab City 21934 Alexandria Egypt

## Abstract

Hypoxia is a distinctive feature of most solid tumors due to insufficient oxygen supply of the abnormal vasculature, which cannot work with the demands of the fast proliferation of cancer cells. One of the main obstacles to limiting the efficacy of cancer medicines is tumor hypoxia. Thus, oxygen is a vital parameter for controlling the efficacy of different types of cancer therapy, such as chemotherapy (CT), photodynamic therapy (PDT), photothermal therapy (PTT), immunotherapy (IT), and radiotherapy (RT). Numerous technologies have attracted much attention for enhancing oxygen distribution in humans and improving the efficacy of cancer treatment. Such technologies include treatment with hyperbaric oxygen therapy (HBO), delivering oxygen by polysaccharides (*e.g.*, cellulose, gelatin, alginate, and silk) and other biocompatible synthetic polymers (*e.g.*, PMMA, PLA, PVA, PVP and PCL), decreasing oxygen consumption, producing oxygen *in situ* in tumors, and using polymeric systems as oxygen carriers. Herein, this review provides an overview of the relationship between hypoxia in tumor cells and its role in the limitation of different cancer therapies alongside the numerous strategies for oxygen delivery using polysaccharides and other biomaterials as carriers and for oxygen generation.

## Abstract 2

Hypoxia is a distinctive feature of most solid tumors due to insufficient oxygen supply of the abnormal vasculature, which cannot work with the demands of the fast proliferation of cancer cells.

## Graphical abstract

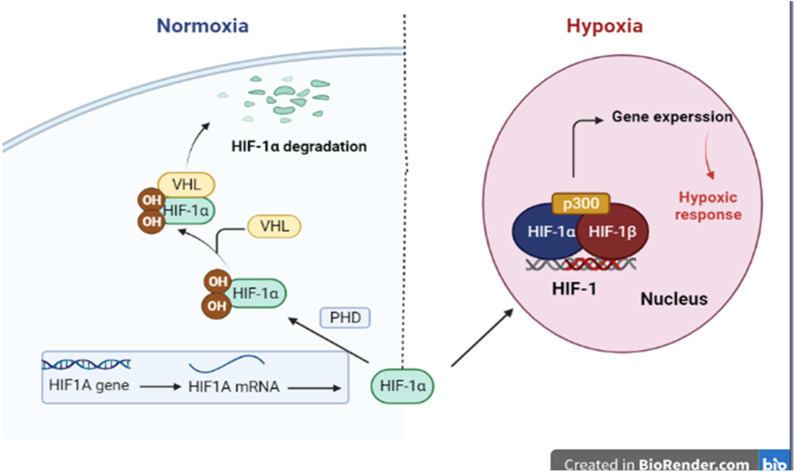

## 1. Introduction

Cancer ranks as the chief cause of death and the main hurdle to significantly improving life expectancy worldwide. Regarding estimations from the World Health Organization (WHO) in 2019, in 112 of 183 countries, cancer is considered the principal cause of death before the age of 70 years and is ranked third in a further 23 countries.^1^ Female breast cancer and lung cancer are the most commonly diagnosed cancers. With a recorded 2.3 million new cases each year, breast cancer accounts for 11.7% of all cancers, followed by lung cancer (11.4%), colorectal (10.0%), prostate (7.3%), and stomach (5.6%) cancers. Lung cancer surpassed breast cancer as the primary reason for cancer mortality, with 1.8 million deaths (18%), followed by colorectal cancer (9.4%), liver cancer (8.3%), stomach cancer (7.7%), and female breast cancer (6.9%).^1^

Tumor cells are abnormal cells that can regenerate rapidly. They are characterized by uncontrolled proliferation, transformation, and migration. Tumor cells resort to unusual metabolic pathways to obtain more energy to meet their needs for cell proliferation and migration, as their metabolism is more enthusiastic than that of normal cells.^2^ As a result of hypoxia, healthy cells are frequently tormented by insufficient oxygen supply, so they transform into cancer cells and the majority of solid tumors originate suddenly and sharply. According to recent studies, tumor hypoxia is a significant barrier to the actual treatment of cancer with immunotherapy and chemotherapy, but not radiotherapy.^3^ Both the intrinsic sensitivity of cancerous cells and the tumor microenvironment influence how cancer responds to chemotherapy. It is well established that tumor hypoxia promotes cancer cells' resistance to radiation and chemotherapy treatments. Moreover, cancer cell migration, growth dynamics, endoplasmic reticulum stress, angiogenesis, and aggressive characteristics are all impacted by hypoxia.^4^ Approximately 60% of advanced solid tumors include hypoxic regions, which is often related to poor survival. Hypoxia-inducible factor (HIF-1α) is a vital regulator of the molecular response to hypoxia. HIF-1α expression is reduced in normal conditions and enhanced by hypoxic conditions. Numerous gene products that influence the regulation of metabolism, cell cycle, angiogenesis, and apoptosis are altered by the activation of HIF-1 expression.^4^

This review explores new trends and strategies for overcoming tumor hypoxia toward enhancing cancer therapy protocols. Oxygen-generating polymeric and non-polymeric biomaterials from the last few decades are reviewed as promising materials for enhancing the efficacy of established cancer therapy protocols *via* reduction of tumor hypoxia.

### 1.1. Induction of solid tumors by hypoxia

**Fig. 1:**
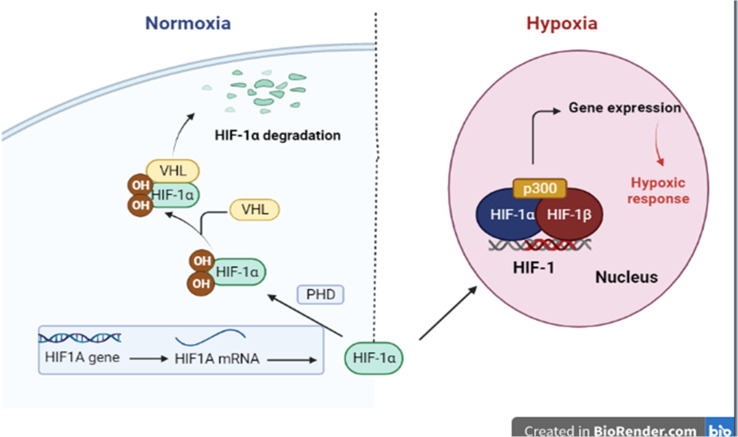
Regulation of HIF-1α stability under normal and hypoxic conditions.

Oxygen-activated breakdown of HIF-1α results in locking genes are enhanced by the environment features with hypoxia properties. Hypoxia is a key factor for inspiring the transition of cobble-stone shaped cells (epithelial cells) to flat-spindle shaped cells (mesenchymal cells), with great potential for metastatic position formation, invasion, and motility proteins; this process is known as epithelial–mesenchymal transition (EMT). Therefore, there is a genuine connection between hypoxia and the proliferation of tumors (Fig. 1).^5^

Under normoxia, the HIF1A gene is rapidly transcribed to HIF1A mRNA, which is then translated to HIF-1α protein. In normal oxygen conditions, the HIF-1α protein is constitutively hydroxylated by the prolyl hydroxylases (PHDs) enzymes, which allows binding of von Hippel–Lindau tumor suppressor protein (pVHL) and ubiquitin ligase, resulting in proteasomal degradation. Under hypoxia, hydroxylation no longer happens, permitting HIF-1α to penetrate the nucleus and form the active HIF transcription complex.

### 1.2. Oxygen-loaded sources in medical applications

Oxygen has a critical role as a signaling molecule and metabolic substrate.^6^ In hypoxic surroundings, human cells need to utilize lactic acid fermentation to yield ATP, which requires fifteen times more glucose to manufacture the same amount of ATP as oxidative phosphorylation. The principal cause of cell necrosis is the depletion of ATP stores,^7^ where hypoxia is the hallmark of ischemic tissue, which has also been shown to promote apoptosis in cells, further stressing the requirement to deliver oxygen. Approaches applied for local oxygen delivery can be mainly divided as mentioned below:

#### 1.2.1. Extracorporeal oxygen delivery system

Oxygen delivery systems are implants that bring oxygen to the implant site. The incorporation of a gas tank within an implant site gives effective supplementation of oxygen to increase islet survival and function within macro devices.^8^ These implantations require daily purging and oxygen replenishment by an external port system.

#### 1.2.2. Oxygen-releasing biomaterials

This technique is based on entrapped oxygen and generates oxygen by chemical reaction.^9^ Oxygen-releasing biomaterials system is an important signaling molecule for monitoring cancer treatment, tissue engineering (TE), and cell proliferation. Numerous techniques have been established to accelerate *in vivo* oxygen delivery and improve the effectiveness of cancer therapies (chemotherapy and radiotherapy) in addition to TE strategies.^6^ Such technologies include peroxide-based materials (oxygen-generating biomaterials) and perfluorocarbon as oxygen carriers.

#### 1.2.3. Peroxide-based biomaterials

Solid peroxides decompose upon exposure to water, releasing oxygen.^10^ Many techniques have been developed recently for releasing oxygen in TE scaffolds. A framework with controlled oxygen release is still required as the targeted tissue can respond to an oxygen shortage in accordance with its needs and prevent the initial burst release. Steady and prolonged release of oxygen from biomaterials provides an ideal environment for cell growth.^11^

As shown in eqn (1), calcium peroxide (CPO) is a solid inorganic peroxide with a high-energy covalent link that readily decomposes and produces oxygen in liquid media:1
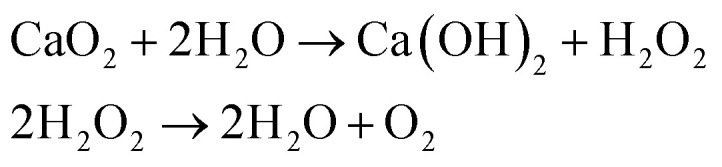


The most popular inorganic peroxide used as an oxygen-releasing source is CPO due to its prolonged release time. Blending CPO into hydrophobic materials can limit the rate of oxygen release in a sustained manner due to the slow dispersion of water into hydrophobic materials and consequently the delayed decomposition of CPO. Additionally, a polymeric matrix's composition, shape, size, and surface chemistry can prevent early burst release and provide adjustable profile release in accordance with the carrier's specifications.^12^ Furthermore, cell micro-carriers have been demonstrated to be a potential strategy for repairing tissues with unconventional shapes, while injectable scaffolds can be used to quickly repair with simple surgical techniques.^13^

An oxygen-releasing antioxidant scaffold was prepared by incorporation of CPO into a polyurethane (PU) scaffold. This scaffold exhibited antioxidant behavior with the sustained release of oxygen are over a period of 10 days. The PU scaffold loaded with CPO reduced the effect of hypoxia *in vitro* and improved cell survival. In an *in vivo* skin flap model, the PU scaffold loaded with CPO (oxygen-releasing scaffold) prevented necrosis.^14^

Recently, a 3-polycaprolactone (PCL) and poly(glycerol sebacate) (PGS) mat loaded with a high concentration of CPO (up to 10%) was utilized as an oxygen source. This composite exhibited the continual discharge of oxygen for several days and meaningfully improved the rate of metabolism due to the decrease of hypoxia in the bone marrow-derived mesenchymal stem cells (BM-MSCs). The manufactured mats showed hints of potential antibacterial efficacy as well. However, for CPO to be used as an oxygen generator, it needs to be made into nanoparticles to ensure good dispersion plus homogeneity when loaded on the nanofibrous mat.^15^

Hydrogen peroxide (H_2_O_2_) is used with caution in biomedical applications due to its limited stability and potential for decomposition to produce reactive oxygen species (ROS) in the biological environment, which can lead to oxidative cell damage. The enzyme catalase, which is present in almost all living organisms, catalyzes the conversion of H_2_O_2_ into water and oxygen and can be utilized to stop H_2_O_2_ buildup.^16^

Due to its capacity to kill bacteria, fungi, and other pathogens, even at low concentrations, H_2_O_2_ is widely used in biomedical applications. It is also present in many oral care products.^17^ Hydrogen peroxide can be capsulated into polyvinyl pyrrolidone (PVP) to produce a PHP complex (PVP–H_2_O_2_ complex) as an oxygen-releasing nanoscaffold.^18^

Our recent study explored the fabrication of poly(methyl methacrylate)-loaded PHP complex (PMMA–PHP complex) platforms as a new model for oxygen-releasing biomaterials with anticancer features. The PHP complex acts as an oxygen generator. The nanofibrous scaffold composed of PMMA + 10% PHP was found to have a decent distribution of PHP (PVP–H_2_O_2_) and exhibited an improvement of mechanical characteristics with uniform nanofibers (NF) as well as prolonged release of oxygen. Depending on the dosage manner, the nanofibers at a concentration of 1 mg ml^−1^ significantly reduced the viability of cells in several cancer cell lines. The viability of cancer cells was reduced to 30%, whereas the normal cells displayed exceptionally safe behavior at the same dose. Additionally, it was shown that the PHP complex, when in the form of a powder, was very hazardous to both healthy and malignant cells, even at low concentrations. However, by embedding the PHP complex in hydrophobic nanofibers, it was possible to avoid the burst release of H_2_O_2_.^5^

Mallepally *et al.* prolonged oxygen release from H_2_O_2_ to 24 hours by using PMMA as the encapsulating material.^19^ Furthermore, Li *et al.* demonstrated prolonged release of oxygen for up to 14 days by encapsulation of H_2_O_2_ into a high-molecular-weight polymer PLGA shell, through which oxygen gradually diffuses.^20^

Sodium percarbonate (SPC) has been extensively used as a source of anhydrous H_2_O_2_ in organic synthesis which then decomposed into oxygen and water.^21^2
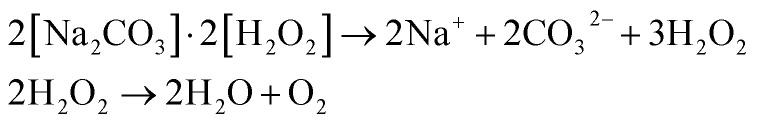


McQuilling *et al.* used SPC to reduce the hypoxic problem on islets (found in clusters throughout the pancreas), commencing with islet separation and continuing 7 days after microencapsulation. They demonstrated that oxygen-producing substances, such as SPO, provide a potentially workable strategy in diabetic patients to replenish oxygen to transplanted islets that are either naked or encapsulated. Although these results revealed that SPO can improve islet viability and functionality, the authors noted that additional work is necessary to control oxygen generation.^22^

#### 1.2.4. Perfluorocarbons (PFCs) based biomaterials

PFCs are highly suitable oxygen carriers for biomedical applications because they consist of fluorinated carbon chains, which display numerous properties. PFCs are inert and thus biocompatible. PFCs have lipophilic properties and self-assemble in aqueous solution, which enhances their stability in mammalian cells. PFCs are miscible with non-polar gases such as NO, CO_2_, CO, and O_2_.^12^ PFCs have high solubility of oxygen and carbon dioxide and allow suitable and controlled release of oxygen to cells, and have been used as oxygen carriers in TE.^6^ PFCs have been used for pancreatic and islet storage prior to transplantation because these substances cannot produce oxygen due to their requiring an oxygen reservoir, which can be troublesome in a closed environment.^22^

## 2. Strategies of using oxygen to overcome tumor hypoxia

There are four ways that oxygen can be used to reduce tumor hypoxia: hyperbaric oxygen therapy, delivering oxygen by carriers to tumors, decreasing tumor oxygen consumption, and generating oxygen *in situ* in the tumor.^23^ These strategies have some advantages and limitations, which will be discussed in the following sections.

### 2.1. Hyperbaric oxygen therapy (HBO)

**Fig. 2:**
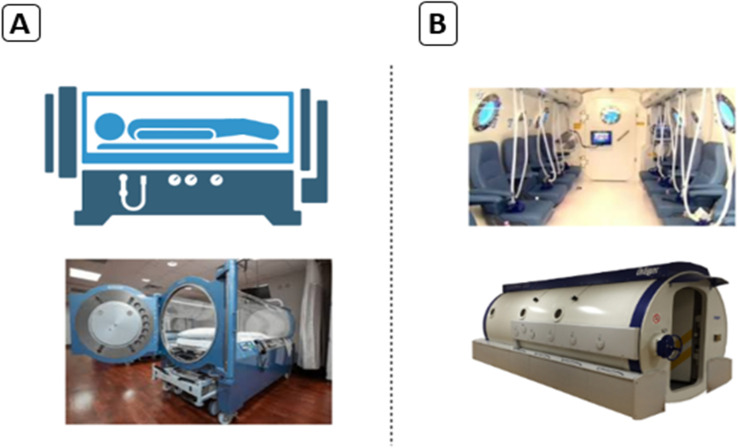
Hyperbaric chambers: (A) mono-place hyperbaric chamber; (B) multi-place hyperbaric chamber.

HBO therapy is termed an effective and safe treatment for different types of cancer.^24^ During HBO therapy, the patient breathes in 100% oxygen, typically at an absolute pressure of 2.5 atmospheres. HBO therapy can enhance oxygen levels in tissues, stimulate angiogenesis, decrease edema, and trigger collagen synthesis.^25^ Consequently, there are several means to implement HBO therapy; the most popular method is to use hyperbaric chambers, either mono-place or multi-place chambers (Fig. 2). Pure 100% oxygen is typically pressurized in mono-place chambers, while a mixture of air with oxygen is normally pressurized in multi-place chambers using an endotracheal tube or face-mask.^26^

Hypoxia plays a critical role in developing tumor drug resistance and the failure of chemotherapy. Overall, there are four detectable mechanisms of drug resistance by hypoxia as follows: (1) hypoxia decreases the intracellular concentration of chemotherapy agents through the accumulation of the drug resistance protein P-glycoprotein, which might push therapeutic agents out. (2) Hypoxia alternates the signaling and metabolic pathways of tumor cells. (3) Hypoxia alters the redox condition of tumor cells. (4) Hypoxia induces mutations and gene instability in cancer cells.^23^

### 2.2. Delivery of oxygen by bio-carriers

**Fig. 3:**
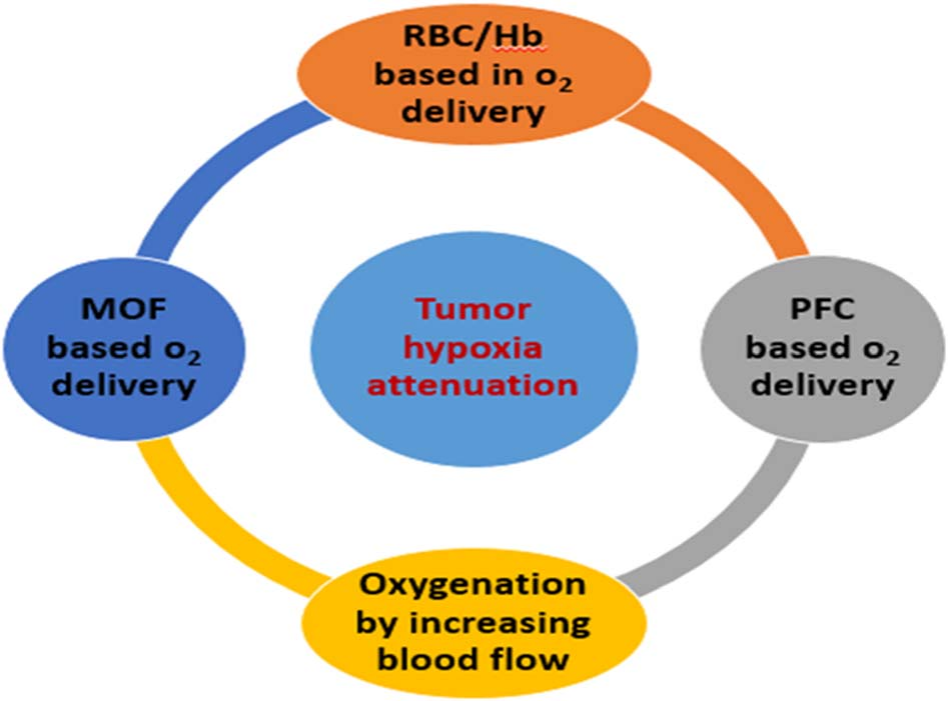
Schematic representation of delivery oxygen by carriers for tumor hypoxia alleviation.

Studies have revealed that there is a critical role for O_2_ carriers in reducing hypoxia to enhance the efficiency of cancer therapies. Artificially carrying molecular O_2_ by innovative nanomaterials to the hypoxic site is one feasible method for increasing the concentration of O_2_. Thus, it reverses the hypoxia and consequently may improve the outcomes of traditional cancer treatments.^27^ There are different types of oxygen-based carriers, including red blood cell (RBC)/hemoglobin (Hb)-based O_2_ carriers, metal–organic framework (MOF)-based O_2_ carriers, and perfluorocarbon (PFC)-based O_2_ carriers, as well as oxygenation by increasing blood flow, as shown in Fig. 3.^28^

#### 2.2.1. RBC/Hb-based O_2_ carriers

Red blood cells are considered good natural candidates as oxygen carriers, owing to their low immunogenicity, easy availability, long blood circulation ability, and biocompatibility. Tang *et al.* developed new technology termed RBC-facilitated photodynamic therapy (RBC-PDT), which can potentially solve the limitations of PDT because of hypoxia.^29^ RBC-PDT uses erythrocytes as a photosensitive carrier, in addition to acting as an O_2_ transporter. Even under hypoxic conditions, RBC-PDT can potentially produce singlet oxygen (^1^O_2_) because photosensitizers are adjacent to a carry an oxygen source. During PDT, RBC has a long blood circulation ability that ensures a high intraluminal concentration of photosensitizers and therefore increases the breakdown of the tumor. They observed that RBC-PDT demonstrated impressive subcutaneous tumor destruction (76%) due to the delivery of both photosensitizers and O_2_.^29^

Hemoglobin (Hb) is a metalloprotein that is responsible for the oxygen-carrying capacity of RBCs. Consequently, Hb is an interesting choice for scientists to create artificial oxygen transporters. Hb has a short blood circulation ability and poor stability in addition to its toxicity, so it needs to be conjugated with suitable carriers.^30^

Hb encapsulated in a liposome system revealed a higher affinity of direct oxygen delivery than RBC and was employed to improve the outcome of radiotherapy (RT) *in vitro* and *in vivo*. Murayama *et al.* proved that the tumor was significantly inhibited by radiation treatment accompanied with intravenous injection of Hb encapsulated in liposomes compared to treatment with radiation and unencapsulated Hb.^31^ Many efforts have been made to conjugate Hb with different types of polymers to create an oxygen delivery system to alleviate hypoxia and hence enhance cancer treatment. Wang *et al.* innovated an oxygen delivery nano-system by encapsulating polystyrene zinc phthalocyanine (PS-ZnPc) into Hb-conjugated polymeric micelles composed of poly(ethylene glycol)-*block*-poly (acrylic acid)-*block*-polystyrene.^32^

Hb-conjugated polymeric micelles produced more singlet oxygen (^1^O_2_) after light initiation and triggered photo-toxicity to cervical cancer cells in comparison with polymeric micelles without Hb conjugation.^32^ Correspondingly, Luo *et al.* enhanced the efficacy of PDT by developing a lipid–polymer hybrid nanoparticle system to mimic RBC seen in mammals.^33^ They complexed Hb with indocyanine green dye (ICG), and subsequently the complex was embedded into lipid–polymer nanoparticles with a DSPE–PEG shell and PLGA core. Tumor-bearing mice injected with Hb–ICG encapsulated nanoparticles showed broad inhibition of tumors due to oxidative damage by generating reactive oxygen species (ROS) from self-supply of oxygen.^33^ Hb-supported oxygen delivery is suitable for avoiding hypoxia accompanying drug resistance. Yang *et al.* encapsulated Hb and doxorubicin (DOX) into a liposome, which they called a DOX–Hb–liposome (DHL) system. In the tumor model, DHL overturned hypoxia and revealed potential antitumor properties compared to the DOX–liposome only without Hb.^34^

#### 2.2.2. MOF-based O_2_ carriers

A highly porous metal–organic framework (MOF) is a crystalline material with a high surface area created by the self-assembly of transition-metal cations/clusters corresponding to multi-dentate organic linkers.^35,36^ MOF as an oxygen carrier has wide attraction for prospective industrial and biomedical applications.^37^ One of the successful efforts was development of an oxygen delivery nanoplatform composed of zirconium(iv)-based MOF (UiO-66) to improve PDT efficacy.^38^ This nanoplatform presented brilliant tumor oxygenation, leading to obviously reduced hypoxia and nearly complete tumor alleviation due to the enhanced PDT efficacy.^38^

An oxygen-loaded pH-responsive multifunctional nano-drug carrier with improved chemo-photodynamic therapy effectiveness was successfully synthesized by Xie and coworkers. The rare earth-doped nanoparticles (NaYF_4_:Yb/Er@NaYbF_4_:Nd@NaGdF_4_) (UC) were engaged for dual-modal up-conversion and MR imaging. Moreover, the UC core–shell structural nanoparticles could efficiently stimulate photosensitizer Rose Bengal (RB) in a mesoporous silica shell (mSiO_2_) for PDT under laser irradiation (808 nm). The shell zeolitic imidazolate framework-90 (ZIF-90) breaks down under acidic conditions, which is the tumor environment, thus promoting the rapid release of DOX and oxygen to overcome tumor hypoxia. This resultant UCNPs-MOF multifunctional oxygen carrier demonstrated an intense antitumor effect both *in vivo* and *in vitro*.^39^

#### 2.2.3. PFC-based O_2_ carriers

Perfluorocarbons (PFCs) are hydrocarbon-conjugated organic compounds in which the hydrogen atoms have totally or partially been replaced by fluorine atoms. Because fluorine has a high electronegativity, PFCs have a remarkable affinity for oxygen.^40^ As a result of their brilliant oxygen affinity, PFCs have the ability to carry roughly twice as much oxygen as red blood cells.^28^ PFCs are commonly administered in the form of an emulsion because of their immiscibility with liquid solutions. Nano-sized PFC emulsions have revealed a defensive role in ischemia as well as preventing pancreatic β-cell hypoxia.^41^ When introduced, the nano-emulsion of PFCs dissolves huge amounts of oxygen in the lung and then releases this oxygen into other tissues. Previously, the ROS creation capability of a photosensitizer (PS) has been enlarged by changing the ration of PFC to PS.^42^ The authors fabricated a sequence of micelles depending on copolymers of PEG, porphyrin, and pentafluoro phenyl, with various ratios of PFC to PS in the core. With an increase in the PFC content, the light-stimulated singlet oxygen production of porphyrin was improved, which subsequently assisted the generation of ROS.^42^ Therefore, PFC as an oxygen carrier has an important role in the improvement of cancer treatment and reducing hypoxia by different routes of PDT,^43,44^ RT^45^ and chemotherapy.^46^

#### 2.2.4. Oxygenation *via* increasing blood flow

Cancer cells feature irregular structures of vessels that are compressed and leaky vascular structures, which tend to impede blood perfusion and elevate interstitial fluid pressure. The abnormal vascularization is the main reason for the restricted diffusion of therapeutic factors inside tumor cells and the hypoxic environment of tumor tissues.^47^ Intermediate-sized nanoparticles (20–40 nm) were found to help with tumor vasculature normalization.^48^ Guo *et al.* used rapamycin as an mTOR inhibitor, which was effective in regularizing the vasculature system of the tumor and enhancing the beneficial impact of cisplatin co-delivery *via* PLGA with a nanoparticle size of 100 nm.^49^

A number of anti-angiogenic therapeutic medications, specifically bevacizumab, sinomenine, combretastatin, and thalidomide, also successfully improved the therapeutic efficiency of numerous nanomedicines *via* fixing abnormal tumor vasculature.^50,51^ A high density of extracellular matrix (ECM) components, like collagen and hyaluronic acid, increases the pressure, which squeezed the tumor's arteries. Cancer-associated fibroblast (CAF) cells are principally responsible for producing these components. Angiotensin inhibitor losartan might decompress the intra-tumoral vessels by deactivating CAFs and reducing the manufacture of stromal collagen/HA. Therefore, losartan therapy improved vascular blood flow, decreased hypoxia in pancreatic and breast cancer simulations, and enhanced chemotherapy.^52,53^

### 2.3. Influence of decreasing oxygen consumption

The microenvironment of tumors is generally characterized by a limited amount of H_2_O_2_ and hypoxia, which result in restriction of the therapeutic efficiency of the combined treatment. Remarkably, modeling and simulation studies have demonstrated that reducing oxygen consumption is obviously more effective at enhancing tumor oxygenation than increasing oxygen delivery.^54^ Consequently, one of the novel alternative approaches for improving the accumulation of oxygen in tumors is reducing oxygen depletion. Recently, anticancer drug doxorubicin (DOX) was successfully loaded onto nanoparticles of copper–metformin (Dox@Cu–Met NPs) to stimulate chemotherapeutic effectiveness and starvation therapy by reducing O_2_ consumption and increasing H_2_O_2_ production, in addition to obstructing the production of ATP. The nano-sheet structure with an appropriate size of Dox@Cu–Met NPs degraded in response to high levels of GSH and the acidic environment of the tumor tissue. Dox@Cu–Met NPs can potentially enhance the concentration of O_2_ in the tumor environment. *In vitro* assay proved that Dox@Cu–Met NPs improves the sensitivity and selectivity of breast cancer cell model (MCF-7/ADR cells) to DOX and encourages the creation of ROS in tumor cells. Meng *et al.* also applied their DOX-loaded NPs in *in vivo* experiments, which confirmed their biosafety and ability to inhibit cancer cell progression. The combination of chemotherapy and nanoparticles can reduce oxygen consumption, resulting in the substantial reduction of tumor proliferation.^55^

### 2.4. Oxygen generation *in situ* in tumor

**Fig. 4:**
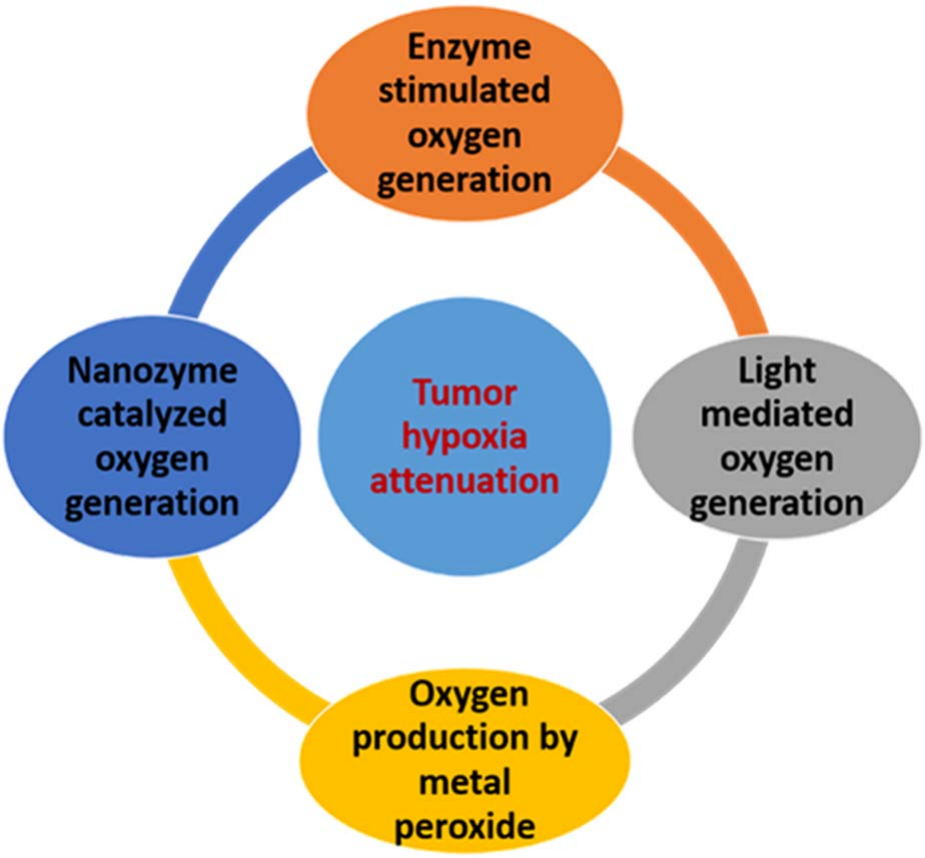
Schematic representation of *in situ* generation of oxygen in the tumor.

High levels of reactive oxygen species (ROS), specifically H_2_O_2_, are generated in tumor cells as the result of a defect of metabolic regulation and uncontrolled proliferation of tumor cells, which might be able to contribute to O_2_ creation to inhibit hypoxia.^56^ Large levels of ROS, primarily hydrogen peroxide (H_2_O_2_), are typically produced by the unchecked growth and malfunction of metabolic regulation in tumor cells. These ROS could be used for *in situ* O_2_ synthesis to combat hypoxia. As shown in Fig. 4, numerous methods have been developed so far to utilize endogenous H_2_O_2_ for cancer treatment and hypoxia relief.

ROS are created as byproducts of cellular metabolism, such as hydroxyl radicals, singlet oxygen, superoxide, and peroxide,^57^ and affect cell homeostasis as well as signaling.^58^ Different human diseases can lead to extreme creation of ROS, which results in oxidative stress and many pathological features.^59^ Cancer cells possess high concentrations of ROS as a result of changes in their metabolic activities.^60^ The elevated intracellular ROS level in cancer cells, therefore, can be considered a cancer-specific stimulus for anticancer drug delivery. Recently, various ROS-responsive drug carriers have been employed to achieve effective anticancer therapy by regulating the release of anticancer drugs in response to the overproduction of intracellular ROS in cancer cells.

#### 2.4.1. Oxygen generation by enzymatic stimulation

The main antioxidant enzyme is catalase, which decomposes H_2_O_2_ into oxygen and water in the body with very high turnover efficiency. In tumor cells, catalase activity is unregulated, resulting in accumulation of H_2_O_2_.^61^ Catalase is an enzyme that exists in the liver and blood of humans. This enzyme consists of four heme (iron-containing organic ring) groups fixed in the structure that are involved in the oxygen conversion processes, although the definite mechanism that explains the catalase function is still unknown.^10^ Chen *et al.* used solvent-diffusion–evaporation and W/O/W double-emulsion method to encapsulate a composite of the antitumor drug cisplatin with catalase into polylactic glycolic acid nanoparticles (PLGA NPs).^62^ The results proved that catalase enhanced oxygen generation, which increased the therapeutic benefits of cisplatin in cancer cells. Another study focused on stimulation of sonodynamic cancer therapy by utilizing catalase-triggered oxygen production. Catalase was loaded into mesoporous organo-silica NPs (MONs) to create a nanoreactor with multiscale catalytic properties.^63^ The hybrid nanoreactor exhibited sustainable generation of oxygen owing to its sensitive activity toward H_2_O_2_. An *in vivo* study was developed to observe the accumulation of catalase@MON NPs in the tumor by enhanced permeability and retention effect (EPR), which mediates the breakdown of endogenous H_2_O_2_ into oxygen. Furthermore, the continual release of oxygen means it can be used as a durable contrast enhancer for ultrasound imaging of tumor cells.^63^ The combination of catalase and a polymeric nanocarrier for O_2_-mediated PDT was developed by Phua *et al.*^64^ In their research, catalase was conjugated with β-cyclodextrin-functionalized hyaluronic acid (HA) loaded with photosensitizer chlorin e6 (Ce6) to form HA–CAT@aCe6 NPs. These nanoparticles revealed high sensitivity and selectivity to breast cancer cells, resulting in depletion of hypoxia *via* the action of catalase, which enhanced the efficacy of PDT.^64^

#### 2.4.2. Oxygen generation by light mediation

Conversion of water and CO_2_ using a light source to give sugars accompanied with oxygen release as a byproduct is termed as a photosynthetic process.^65,66^ Various studies have utilized water splitting as a source of oxygen production. Recently, water splitting has been used for oxygen regeneration by two-photon radiation of iron-doped carbon nitride (Fe–C_3_N_4_) to reduce tumor hypoxia.^67^ (Fe–C_3_N_4_)NPs were loaded with a photosensitizer, which was a complex of ruthenium(ii) tris(bipyridyl) (Ru(bpy)_3_^2+^). Exposure to two-photon laser activated Ru(bpy)_3_^2+^ to produce singlet oxygen (^1^O_2_) and Fe–C_3_N_4_ was initiated to split water for oxygen supply in the meantime. The nanoparticles were shown to reduce the tumorous hypoxia and improve the efficiency of PDT after nanocomposite accumulation by improving the EPR effect.^67^

Zhou *et al.* developed a biological system based on *Chlorella pyrenoidosa* surrounded by calcium alginate, which they termed ALGAE (autotrophic light-triggered green oxygen-affording engine).^68^*Chlorella* is a photosynthetic green algae and single autotrophic cell possessing photosynthetic pigments in its chloroplasts, which makes it a good candidate as a biocompatible oxygen resource.^69^ The ALGAE were fixed around the cancer cells in a slightly invasive way and could be reserved for an extended period for oxygen supplying because the *Chlorella* was protected *via* calcium alginate from scavenging by macrophages. A source of light energy was necessary for *Chlorella* to generate oxygen during PDT mediated by ALGAE treatment. The ALGAE stimulated oxygen generation through energy transformation and water splitting, and thus the process is ecofriendly and biosafe. ALGAE could surround cancer cells and produce abundant oxygen *in vivo* after irradiation.

During hypoxia-resistant PDT induced by ALGAE, light is not only one of the elements of PDT, but also the source of energy for *Chlorella* to generate oxygen. The oxygen is generated with light triggering by ALGAE through water splitting and energy transformation, and the overall process is economical and environmentally friendly. ALGAE can stay around tumor tissues and generate copious oxygen *in vivo* after receiving irradiation, and simultaneously the photosensitizer produces enough singlet oxygen, thus destroying the cancer cells and improving the efficiency of PDT.^68^

#### 2.4.3. Oxygen generation *via* metal peroxides

Metal peroxides (MO_2_), including MgO_2_, CaO_2_, CuO_2_, ZnO_2_, and BaO_2_, can provide a strong supply of oxygen. MO_2_ results in the slow release of oxygen in water with strong oxidation in acidic conditions *via* its breakdown products (H_2_O_2_).^70^ Recently, MO_2_ has been used as an O_2_-creating material and applied to the manufacture of a tumor theranostics nanoplatform, which can control the tumor microenvironment (TME) to form a fresh work environment for those treatments whose efficiency is restricted by the TME.

Bu *et al.* fabricated transferrin-modified MgO_2_ nanosheets (TMNSs), which effectively respond to the acidity of TME. MgO_2_ reacts rapidly with H^+^ to produce H_2_O_2_ and break down the structure of transferrin on the nanosheets.^71^ Subsequently, transferrin releases the ferric ions (Fe^3+^) and creates cytotoxic hydroxyl radicals (˙OH) by the Fenton reaction. The TMNSs generate a large concentration of H_2_O_2_ and release ˙OH, which has the capability to destroy tumor cells, while TMNSs in normal cells (alkaline condition) produce a small amount of H_2_O_2_, which is decomposed by catalase. The results confirmed the high selectivity of TMNSs for tumor cells.^71^

Lin *et al.* successfully developed PVP-modified ZnO_2_ NPs and fixed them with paramagnetic Mn^2+^ ions by the cation exchange method.^72^ In this nano-system, ZnO_2_ disintegrates into H_2_O_2_ and Zn^2+^ in the acidic environment of the tumor. One of their valuable observations is that Zn^2+^ enhanced the mitochondrial creation of ROS by preventing the electron transfer chain, which was proved also by other studies.^73,74^ The exogenous release of ROS combined with the endogenous generation led to a significant tumor-killing effect.^72^

Jiang *et al.* constructed nanoplatform mediated CDT by fabricating hybrid CaO_2_ and Fe_3_O_4_ NPs with hyaluronic acid (HA) as a stabilizer and a NIR fluorophore label in the form of CaO_2_–Fe_3_O_4_@HA NPs.^75^ The nanoplatform possessed a great capability for self-supplying H_2_O_2_ and generating ˙OH in the acidic conditions of the TME with delectable stability under physiological conditions. It also revealed great selectivity and sensitivity to tumor cells with an inhibiting rate of up to 70% by CDT, while being safe to normal cells.^75^

#### 2.4.4. Oxygen generation by catalyzed nano-enzymes

Nanomaterials with enzyme-like catalytic properties are called nanozymes.^76^ These manufactured enzymes are the focus of numerous studies due to their desirable properties, great stability, robustness and ease of fabrication.^77^

Feng *et al.* innovated novel TME-modulated nanozymes using tin ferrite SFO (SnFe_2_O_4_) for mediating phototherapy (PT), photothermal therapy (PTT) and chemotherapy (CT).^78^ The synthesized SFO nanozymes possessed both CAT- and GSH-like activities. The SFO nanozymes enhanced the PT efficiency by activating H_2_O_2_ to create oxygen to inhibit tumor hypoxia. Meanwhile, the SFO nanozyme in the TME efficiently effected CT by mediating H_2_O_2_ to generate (˙OH) *in situ* combined with the depletion of GSH to release the antioxidant ability of the tumors. Moreover, the SFO nanozyme irradiated with 808 nm laser exhibited an outstanding phototherapeutic effect on account of the improved PTT efficiency and excellent free radical generation performance.^78^

Recently, a multi-nanozyme design was constructed based on polymeric HA-mediated CuMnO_*x*_ NPs (CMOH) overloaded with indocyanine green (ICG) to form HA–CuMnO_*x*_@ICG nanocomposite (CMOINC) with highly efficient ROS production, hyperthermia, oxygen self-evolving function, and GSH reduction capability for attaining hypoxic tumor therapy.^79^ The CMOH nanozyme system showed oxidase- and peroxidase-like activities, which could powerfully initiate H_2_O_2_ or O_2_ to produce superoxide radicals (˙O_2_^−^) or hydroxyl radicals (˙OH) in the TME, enriching the oxidative stress of the tumor. CMOINC was shown to be highly effective at generating ^1^O_2_ and *in situ* hyperthermia under light irradiation.^79^ Different nanozymes revealed high efficiency for integration with multiple treatment modalities. Thus, this strategy may provide a new dimension for the design of other TME-based anticancer strategies.^28^

### 2.5. Oxygen delivery-based polymeric biomaterials

Polymeric materials have become extensively widespread in numerous biomedical applications. Biomedical polymers can be classified into natural and synthetic polymers. Natural polymers like silk, alginate, cellulose, gelatin, agar, fibrin, collagen, pectin, and chitosan are of wide interest for medical applications, owing to their nontoxicity, biocompatibility with the human body, biodegradability, and ability to accelerate cell proliferation. However, they have the critical disadvantages of lack of mechanical strength and inappropriate degradation rate.^80^ Synthetic polymers (like polyacrylonitrile, polyurethanes, polyamides, polycarbonates, polyvinyl alcohol, poly caprolactone, polyvinyl chloride, and polyesters) are applied for regenerative medical applications owing to their desirable mechanical properties, good integration with the neighboring tissue, ability to enhance cell adhesion, differentiation, and good cell migration.^81,82^ Biomedical polymers can be divided into different dimensional classes, such as:^83^ (a) 0D, such as nano/micro spheres, shells or capsules, (b) 1D, such as fibers or suture materials, (c) 2D, such as coatings or films, (d) 3D solids, like porous or printed polymers, and (e) 3D soft, like gel or foam.

#### 2.5.1. Carriers based on natural polymers

Natural polymers demonstrate poor mechanical properties, but they possess respectable biocompatibility and desirable bioactivities, which make them promising candidates for oxygen delivery. Herein, we briefly discuss some recently natural polymers that have been used as oxygen carriers for the enhancement of cancer treatment.

Silk is composed of proteins, including the core protein of fibroin (70–80%) and adhesive proteins termed sericin (20–30%).^84^ It is produced from the cocoons of the larvae of silkworms^85^ or other insects like spiders.^86^ Silk possesses notable mechanical properties, biocompatibility, and flexibility, and remarkable degradation rates, both *in vitro* and *in vivo*.^87^ It has been permitted by the Food and Drug Administration (FDA) for utilization in the creation of sutures as a biomedical material in surgery.^88^ Arumugam *et al.* used an electrospun gold–silver nanoparticle-loaded-nanofiber scaffold of silk fibroin (SF) and cellulose acetate (CA/SF/Au–Ag) for anticancer applications. The silk in addition to cellulose acetate acted as the stabilizing agent for silver and gold ions with better biocompatibility. The fabricated scaffold had needle-shaped morphology with diameter of 86 nm and the Ag and Au nanoparticles were dispersed onto the fiber scaffold with an average size of 53 nm and 17 nm, respectively. Moreover, it powerfully activates the cytotoxic effects against MDA-MB-231 and MCF-7 human breast cancer cells with a potential IC_50_ value. They found that the SF/CA/Au–Ag composite nanofiber scaffold is a promising material for anticancer applications.^89^

In another study, Yang *et al.* developed a platform of SF loaded with MnO_2_ NPs and indocyanine green (ICG) as a photodynamic agent in addition to doxorubicin (DOX) as a chemotherapeutic agent that can be customized in the form of SF@MnO_2_/ICG/DOX (SMID) nano-system.^90^ This nano-system exhibited high reactivity with H_2_O_2_ in the TME, which was broken down into oxygen to improve PDT. They also proved that the SMID nano-system had a distinctive effect for PTT as the result of its strong photothermal response to NIR (near-infrared) radiation and stably conjugated ICG. Moreover, their animal studies confirmed that the SMID nano-system obviously enhanced tumor alleviation through the combination of PDT, PTT, and chemotherapy with low toxicity. SF-based bio-nanomaterial, therefore, represents a promising carrier for oxygen to reduce hypoxia of the TME and improve cancer therapy.

Alginate is a promising inert natural polymer that is easily extracted from algae, generally considered for its capability to facilitate drug delivery *in vivo* with unique features,^91^ like biodegradability, muco-adhesion, biocompatibility and biosafety.^92^ Moreover, alginate has been applied as a bio-carrier for liposomes to capture hydrophilic chemotherapeutic agents and assist their transport to the targeted cell.^93^ Alginate-based biomaterials are extensively applied in drug delivery, wound dressing, and tissue engineering.^94^ The combination of CaO_2_ and catalase encapsulated into an alginate solution was successfully fabricated as a source of oxygen release.^95^ Huang *et al.* established that implantation of alginate-encapsulated CaO_2_ close to the tumor area stimulated the decomposition of CaO_2_, when reacted with H_2_O, into H_2_O_2_ and calcium hydroxide, which then decayed to generate molecular oxygen. Subsequently, oxygen-generating depots in TME improve the response to chemotherapeutic administration of DOX.^95^

Cellulose and its derivatives have been utilized efficiently as a drug delivery system for numerous types of drugs.^96–98^ Both cellulose nanocrystals (CNCs) and cellulose nanofibrils are represented as important biocompatible and biodegradable nanomaterials with good biosafety.^99,100^ Targeting the TME has been the focus of various studies involving cancer therapeutics and epigenetics. Bhandari *et al.* developed oxygen nanobubbles (ONB) by encapsulating oxygen into nano-size carboxymethyl cellulosic nanobubbles for modifying the hypoxic area of tumors to obstruct tumor growth.^101^ ONB were able to significantly deplete tumor progression and accelerate survival rates in animal models. ONB reprogramed tumor suppressor- and hypoxia-associated genes like PDK-1 and MAT2A, preposition are aiding as an ultrasound contrast agent.^101^

Gelatin is a hydrophilic natural polymer extracted from the collagen in the bones and skin of animals, and it has been utilized as drug delivery carrier, a food additive and a scaffold for TE because of its elasticity and viscosity, as well as its specific temperature-controlled gelation behavior.^102^ In addition, above the specific gelation temperature, gelatin can be developed into an applicable ecofriendly film by solvent evaporation.^103^

Plasma-treated liquids were recently established to have discerning features for inhibiting cancer cells and have attracted interest for use toward plasma-based cancer therapies. Labay *et al.* showed that reactive oxygen and nitrogen species (RONS) can be generated by atmospheric pressure plasma jets in liquids and biological systems.^104^ They used gelatin solution to store RONS produced by atmospheric pressure plasma jets to design an innovative biomaterial for cancer treatment. The quantity of RONS created in gelatin is significantly upgraded with respect to water, with concentrations of H_2_O_2_ and NO_2_^−^ between 2 to 12 times greater for the longest plasma treatments. Plasma-treated gelatin showed the discharge of RONS to a liquid media, which encouraged effective elimination of bone cancer cells with high selectivity. The results set the basis for the approach for developing hydrogels with a high capacity to deliver RONS to tumors.^104^

An alternative approach for oxygen supplementation was revealed by Mizukami *et al.*, where they fabricated HepG2 spheroids containing GMS (gelatin microsphere) by first manufacturing 37 μm GMS by water–oil emulsification then freeze drying them; HepG2 hepatocyte cells were then incubated with GMS at several mixing ratios in agarose gel-based micro-wells.^105^ They combined GMS into the core of the HepG2 human hepatocyte spheroids to permit oxygen to reach the spheroid core. HepG2 cells in the GMS/HepG2 spheroids were more highly oxygenated than those in the GMS-free spheroids. The viability of HepG2 cells in the spheroids was increased by GMS incorporation and further the CYP1A1 of activity of the HepG2 cells was enhanced to metabolize 7-ethoxyresorufin. In addition, mRNA expression of the CYP1A1 gene was significantly affected by GMS integration. The results designated that combining GMS with HepG2 spheroids increased the bio-viability of the cells and the CYP1A1 metabolic activity.^105^

#### 2.5.2. Carriers based on synthetic polymers

The use of synthetic polymers as oxygen carriers for enhancement of cancer treatment is still limited. One study developed a novel synthesized polymer as a singlet oxygen carrier for hypoxic tumor treatment. The polymeric carrier was composed of hydrophilic polyethylene glycol (PEG), 1,4-dimethylnaphthalene (DMN), a reserved modifiable disulfide group, and a phosphorescent iridium(iii) complex.^106^ Lv *et al.* innovated a synthetic polymer as a carrier for ^1^O_2_ to overcome tumor hypoxia and its limitation of PDT. This carrier might be applicable for delivering singlet oxygen inside cancer tissue without oxygen consumption. The generation of ^1^O_2_ was activated by the photothermal (PT) effect of Au NRs@PEG under NIR light radiation, realizing photothermal-controlled oxidative damage and photothermal damage to cancer cells.^106^

Liu *et al.* examined the effects of low oxygen conditions on cell survival and oxygen generation in a synthetic system. They established a 3D system using calcium peroxide (CaO_2_) and poly(lactic-*co*-glycolic acid) (PLGA) microspheres dispersed in a hydrogel. The synthetic oxygen-generating system was tested under hypoxic conditions using stem cells *versus* controls to examine its potential for oxygen generation in a period of up to 21 days. The hydrogel gave prolonged oxygen release, protected the microspheres, and enhanced cell adhesion and cell proliferation in a flexible manner. The system produced oxygen and supported cell growth, which is also predicted to stimulate stem cell growth and survival after implantation.^107^

## 3. Summary of our recent findings

The current research in regenerative medicine and tissue engineering for cancer treatment is focused on developing effective, low-cost, bioactive, and advanced biomaterials for targeting the therapeutic agents and curing tumor hypoxia. Our recent study investigated the fabrication of PMMA–PHP complex nanofibrous platforms and the generation of oxygen nanobubbles as a novel model of biomaterials possessing anticancer properties.

### 3.1. Fabrication of oxygen-releasing nanofibrous scaffold

#### 3.1.1. Synthesis of the PHP (PVP : H_2_O_2_) complex and PMMA/PHP scaffold as an oxygen source

**Fig. 5:**
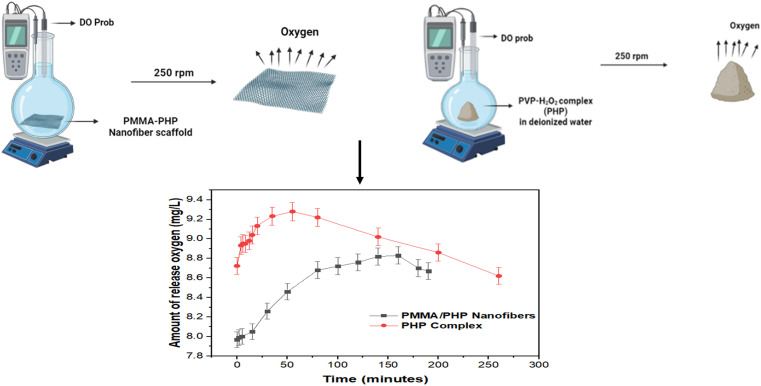
DO release concentration in deionized water for PHP complex and PMMA/PHP nanofibrous scaffold.

The utilization of the PHP (PVP : H_2_O_2_) complex as an oxygen source previously proved the importance of oxygen in the treatment of cancer. The different ratios of PVP : H_2_O_2_ used to gain the complex using procedures were clearly described previously.^5^ Notably, the yield of the developed complex climbed with increasing H_2_O_2_ ratio up to 1 : 1.5, at which point it started to decrease. In contrast to the yields for the other investigated ratios, a 1 : 1.5 ratio of PVP : H_2_O_2_ provided the optimal yield of the complex of 66%. The PMMA + 10% PHP scaffold was reported to have the highest mechanical performance with smooth nanofibers, as shown before by examining its swelling and degrading properties. It also had excellent dispersion of the PHP complex. The concentration of oxygen released from both the PMMA scaffold and the PHP complex is presented in Fig. 5. As shown in the graph, the PHP complex in powder form released oxygen in a faster manner compared to the oxygen released from the PMMA scaffold loaded with the PHP complex. These results matched with those reported by Ahmed and his coworkers for their research based on measuring dissolved oxygen (DO). The DO increased over time (for 120 minutes) in the case of oxygen nanobubbles (ONB), while it decreased when air nanobubbles (ANB) were dispersed into deionized water.^108^

#### 3.1.2. *In vitro* and *in vivo* cytotoxicity evaluation for PHP complex and PMMA/PHP scaffold

**Fig. 6:**
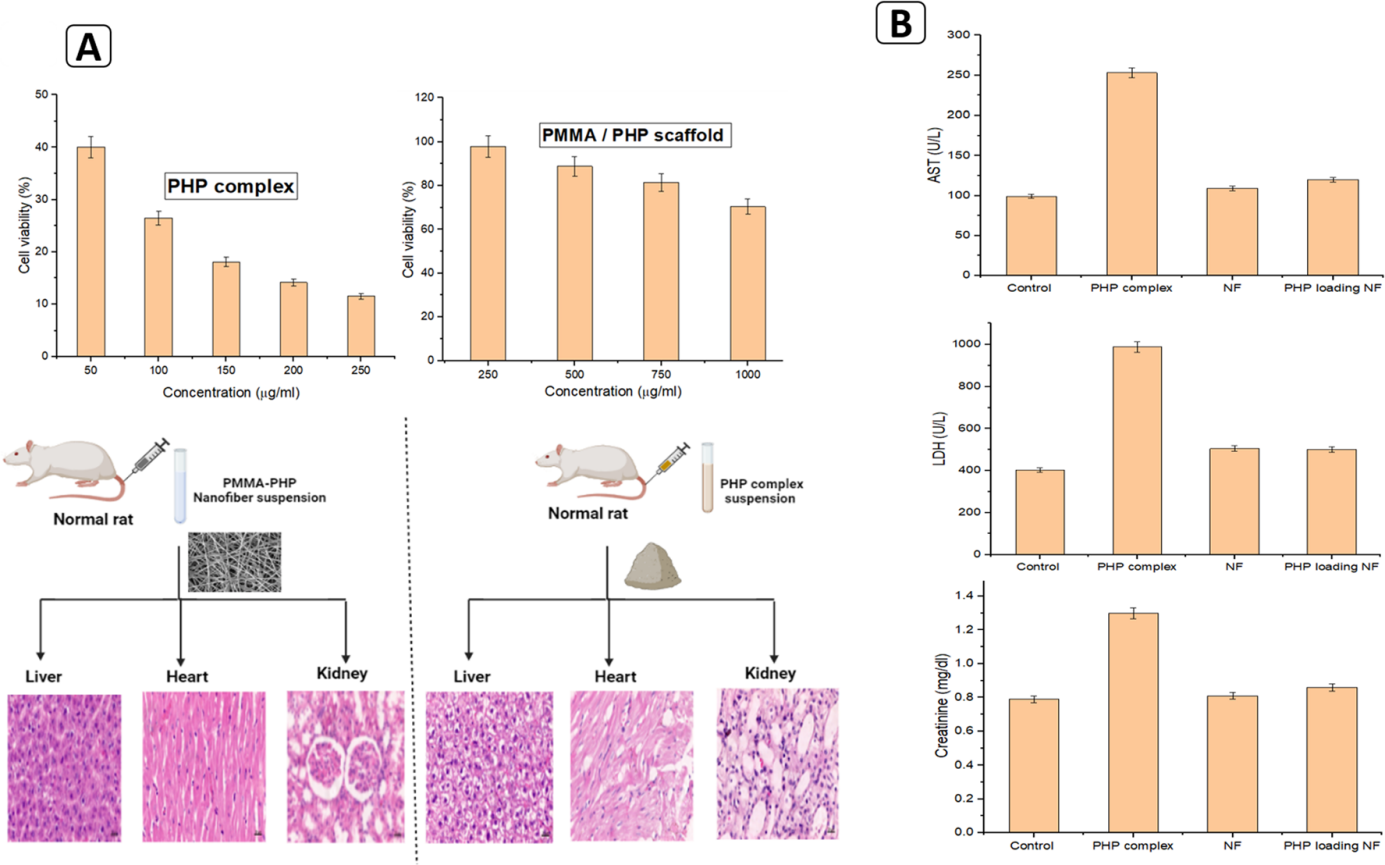
Cytotoxicity assessment investigation for the PHP complex and the PMMA/PHP nanofibrous scaffold (A) *in vitro* (cell lines) and (B) *in vivo* (mice).

In our *in vitro* study, the nanofiber scaffold dose (1 mg ml^−1^) significantly reduced the viability of many cancer cells, reducing it to 30%; yet the same dose demonstrated extremely safe behavior on normal cells. It was evident that the PHP complex as a powder was highly toxic to both healthy and malignant cells, even at low concentrations; however, the toxicity of the PHP complex was reduced by loading it onto hydrophobic nanofibers to prevent the burst release of H_2_O_2_, as described in Fig. 6(A). The main challenge toward successful cancer chemotherapy is toxicity against normal tissues. Consequently, reducing the adverse toxic effects of chemotherapeutic drugs in healthy tissues is very important. To investigate the effects of the fabricated nanofibers on healthy tissues, the PHP complex, PHP-loaded NF, and the unloaded nanofiber scaffold were injected into mice, and biochemical indicators of tissue injury and histopathological changes in the heart, liver, and kidney were investigated. Four days after injection of the PHP complex, PHP-loaded NF, the unloaded nanofiber scaffold, and saline (as a control group), the toxic effects on organs were estimated by detection of serum levels of LDH, AST, and creatinine as biochemical parameters for the heart, liver, and kidney, respectively. High toxicity on normal tissue was recorded when treating the normal cells with PHP, as observed in the results of the *in vitro* cytotoxicity study, but the treatment with fabricated PHP-loaded nanofibers showed no toxic impact on the normal tissue, even at a high concentration. It was found that, PHP complex, PHP-loaded NF, and the unloaded nanofiber scaffold were injected into mice, and biochemical indicators of tissue injury and histopathological changes in heart, liver, and kidney in mice were investigated as shown in Fig. 6(B). The mice treated with fibers had no changes in the biochemical parameters and no histological changes were recorded compared to the control group.

#### 3.1.3. *In vitro* and *in vivo* anticancer activity evaluation for PHP complex and PMMA/PHP scaffold

**Fig. 7:**
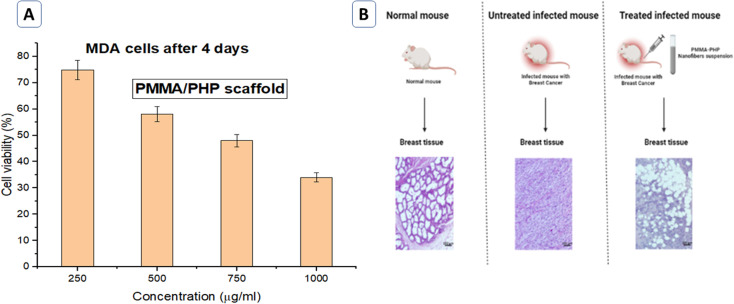
(A) Anticancer activity evaluation *in vitro* (MDA cells). (B) Photomicrographs of H&E stained breast tissue for −ve and +ve control mice and mice treated with and PHP-loaded NF.

The anticancer effect of the PMMA/PHP scaffold was studied on a breast cancer cell line (MDA). Following 4 days of incubation with the PMMA/PHP nanofibrous scaffold, the vitality of the cells was evaluated using the MTT assay, as shown in Fig. 7(A). According to the results, the PHP complex loaded onto the PMMA nanofibers appears to have a clear anticancer effect on the MDA cells, which was reduced to 30%. The presence of the PHP complex, which released oxygen as an inhibitor for cancer cell development, was responsible for the majority of the anticancer effect of the nanofibrous scaffolds; this discovery was corroborated by prior observations by Sletta *et al.*,^109^ who showed that after receiving hyperbaric oxygen therapy, there was significant inhibition of tumor growth, particularly in the BT-474 breast cancer cell and the human MDA-MB-231 cell line.

Clear morphological alterations are often present in the tumor's core, which is significantly observed in a positive control mouse, which also showed multinuclear tissue in addition to increased vessel density in the tumor with dense leukocyte infiltration. The tumor's malignant cells differ noticeably in terms of their cellular and nuclear structure, with vesicular nuclei and obvious nucleoli; this result is in agreement with those reported by Zheng *et al.*^110^ Moreover, the mice treated with PHP-loaded NF showed a decrease in the percentage of necrosis and mitosis compared to the positive control mice, as shown in Fig. 7(B). The PMMA–PHP complex nanofibrous platform was suggested as a potential biomaterial for cancer treatment based on our findings.

### 3.2. Creation and characterization of oxygen nanobubbles (ONBs) as an oxygen source

For many years, medical professionals have used nanobubbles (NBs), vesicles with a spherical shell and a core, as ultrasonic (US) contrast agents.^111^ Due to their miniscule size, US contrast agents are commonly referred to as “nanobubbles”. A non-invasive real-time molecular imaging technique based on the optical absorption of tissues, photoacoustic imaging, also uses NBs as contrast agents.^112^ Due to their nanoscale size, which increases their penetration through cells, nanobubbles have been studied for diagnostic and therapeutic applications. When high US frequencies are used, nanobubbles have similar echogenic characteristics to microbubbles and are retained in tumors for longer periods of time than microbubbles, according to Yin *et al.*^113^ NBs can be created using a variety of techniques, including sonication, agitation, and the use of microfluidic devices to deliver oxygen to precise locations. Although flexible and easy to construct and position for applications, phospholipid shells are hindered by their short half-life. Incorporating surfactants like PEG has been done in a variety of ways to stabilize phospholipid bubbles at the nanoscale. A previous study used oral administration of lipid-based oxygen NBs for enhancing the tumor response to sonodynamic therapy (SDT), where the results showed a significant drop in the rate of tumor growth in the groups treated with oxygen NBs for either 5 or 20 minutes before SDT.^114^

**Fig. 8:**
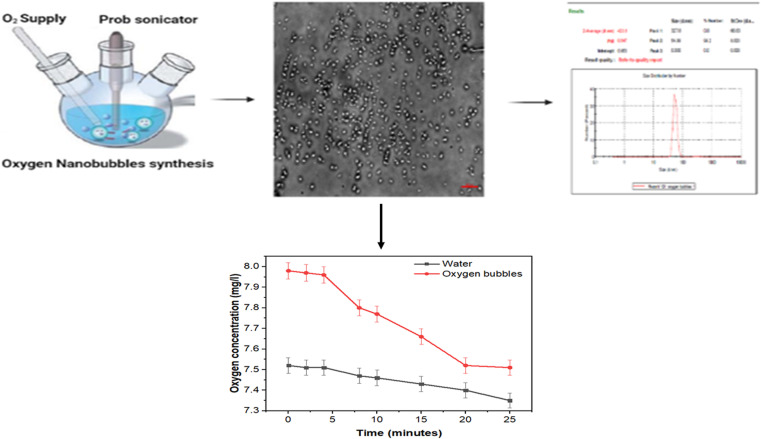
Fabrication and characterization of ONBs.

In our recent study, NBs were prepared from a mixture of 25.2 mg of DPPC (1,2-dipalmitoyl-*sn-glycero*-3-phosphocholine) and 8.4 mg of DPSE–PEG2000 (1,2-distearoyl-*sn-glycero*-3-phosphoethanolamine-*N*-[methoxy(polyethylene glycol)-2000]) in a 75 : 25 molar ratio were dissolved in a 1 : 1 mixture of methanol and chloroform inside a three-neck conical flask, as shown in Fig. 8. A thin-layered lipid film was obtained by drying in a hot air vacuum. The lipid layer was then re-suspended into 10 ml of PBS with 10% glycerol to get the final concentration of 3.36 mg ml^−1^ of lipids, which was then sonicated at 50 °C using a water bath until a milky suspension was formed. Furthermore, the suspension was sonicated using probe-sonication with pulsed mode at 190 W for 5 min in the presence of an oxygen supply to fabricate nanobubbles.^111^ The size distribution and zeta potential of the oxygen nanobubbles were determined using a zeta-sizer (Malvern, USA).

The obtained results showed that the spherical NBs were created clearly with moderate stability and negative charge due to use of a mixture of phospholipids as the shell. Moreover, the stability was enhanced by adding glycerol, which establishes an equilibrium between the shell and the gas core. The oxygen was released in a continuous manner for up to 20 minutes compared with the same volume of water, which was 30 ml. This estimation ensured the successful fabrication of oxygen nanobubbles with moderate stability and further experiments are required to improve their stability.

## 4. Conclusions

The limitation of different cancer therapies is strongly dependent upon tumor hypoxia. There have been numerous attempts through different approaches to reverse hypoxia of cancer cells to date; however, there is no treatment approved by the FDA to reverse tumor hypoxia. Recently, two novel products have been reported to get rid of tumor hypoxia, however two products are currently still undergoing further clinical trials. The first treatment is designed to increase the diffusion of oxygen. The second treatment is fluorocarbons with low boiling points, which have the capability to act as an oxygen delivery system with high efficiency. Prospective, randomized, and placebo-controlled clinical trials will be essential to reveal the efficacy of oxygen as a novel supplement for overcoming tumor hypoxia. Moreover, to establish oxygen as an alternative method for enhancing cancer treatment, there are further issues that urgently need to be explored, such as focusing on all the factors that lead to hypoxia in normal cells, converting them to tumor cells, studying if the hypoxia in tumor cells is reversible or irreversible, understanding the mechanism of oxygen inside the tumor cells and the catalase function for decomposing the H_2_O_2_ into water and oxygen, and optimizing the conditions for enhancing cancer treatment using oxygen.

## Conflicts of interest

The authors report no conflicts of interest in this work.

## Supplementary Material
